# Supplementary material for: GABAergic neurons can facilitate the propagation of cortical spreading depolarization: experiments in mouse neocortical slices and a novel neural field computational model
Source: PLoS Comput Biol. 2025 Jun 4;21(6):e1013099. doi: 10.1371/journal.pcbi.1013099 (PMC12136302; doi:10.1371/journal.pcbi.1013099)
Supplement: S1 Table — Data used to calculate the means (PDF) [file pcbi.1013099.s001.pdf]

Data used to calculate means±SEM

Fig. 1F. CSD propagation speed measured with IOS; WT slices 2.13±0.06 mm/min; VGAT-ChR2-tdtomato slices 2.35±0.12 mm/min)

WT	VGAT-ChR2
1.841064	2.767976
2.012543	2.143632
1.522406	1.753821
2.079298	1.398466
2.348558	1.877864
1.918	1.937448
1.996533	2.77038
3.070135	2.562717
2.173527	1.960157
2.329293	2.693475
2.108559	2.458405
2.267681	2.130525
2.01671	3.548742
1.995789	2.340408
2.061656	2.44293
2.09157	2.025012
1.863165	2.793228
2.71908	2.713596
2.464514	
2.219013	
2.154198	
1.918239	
1.974883	
1.928578	

Fig. 1G. CSD propagation speed measured considering the time lag of the onset of the DC shift and the distance between the two electrodes; WT slices 2.24±0.08 mm/min; VGAT-ChR2-tdtomato slices 2.31±0.11 mm/min.

WT	VGAT-ChR2
1.90875	2.72086
2.08622	2.10714
1.59876	1.72397
2.15524	1.37466
2.43425	1.8459
1.98861	1.90447
1.9827	2.72322
3.77511	2.5191
2.25383	1.92679
2.41453	2.64763
2.1858	2.41656
2.64721	2.09426
2.09115	3.48834

2.06946	2.30057
2.13749	2.40135
2.16805	1.99054
1.73424	2.74568
2.81876	2.66741
2.55552	
2.30115	
2.23312	
1.98755	
2.04777	
2.25908	

Fig. 2B. CTRL 2.22±0.06mm/min; ISO 2.05±0:06 mm/min; GBZ 3.70±0.09 mm/min; LIGHT 2.31±0.11 mm/min; GBZ+LIGHT 5.04±0.15 mm/min.

CTRL	ISO	GBZ	LIGHT	GBZ+LIGHT
1.841064	1.887471	3.391774	1.62	6.52329
2.012543	2.016162	3.27181	2.06	4.848558
1.522406	2.209218	3.226883	2.46	3.549795
2.079298	2.037246	3.679213	2.13	5.191932
2.348558	2.22159	4.247786	2.38	4.802226
1.918	2.440875	3.742684	2.82324	4.94763
1.996533	2.309523	4.010217	1.707276	4.224492
3.070135	1.7445	3.872514	1.943523	5.449713
2.173527	1.780341	3.854385	3.652767	4.964364
2.329293	1.875357	4.147774	2.588385	6.908964
2.108559	2.075463	3.386472	2.376925	5.670372
2.267681	2.145285	4.004358	2.850078	5.293488
2.01671	1.903383	3.313755	1.830774	5.169402
1.995789		3.623931	3.093978	5.169096
2.061656			2.751588	5.44584
2.09157			2.699442	5.008884
1.863165			1.735863	4.243704
2.71908			1.899042	4.442562
2.464514			1.771606	4.30593
2.219013			1.852208	4.797066
2.154198			1.941603	4.976064
1.918239			2.169848	4.353126
1.974883			3.187095	5.669262
1.928578			2.562717	
2.767976			1.667985	
2.143632				
1.753821				
1.398466				
1.877864				
1.937448				

2.77038				
2.562717				
1.960157				
2.693475				
2.458405				
2.130525				
3.548742				
2.340408				
2.44293				
2.025012				
2.793228				
2.713596				

Fig.3B. CSD propagation speed in control before blue light (2.20±0.11 mm/min) and during blue light (2.31±0.11 mm/min).

**Before blue light**	**During blue light**
1.67	1.62
1.83	2.06
1.69	2.46
1.33	2.13
1.54	2.38
2.719236	2.82324
2.13291	1.707276
2.371724	1.943523
2.917028	3.652767
2.22076	2.588385
2.474076	2.376925
2.669064	2.850078
2.726742	1.830774
3.577962	3.093978
2.237406	2.751588
2.16267	2.699442
2.636478	1.735863
2.026176	1.899042
1.77321	1.771606
1.346184	1.852208
1.829235	1.941603
1.635207	2.169848
2.525438	3.187095
1.968384	2.562717
2.960412	1.667985

Fig.3D. CSD propagation speed in the presence of GBZ before blue light (4.07±0.15 mm/min) and during blue light (5.17±0.15 mm/min).

**Before blue light**	**During blue light**
5.2451475	6.52329
4.49166	4.848558
3.154842	3.549795
4.28854	5.191932
4.395072	5.293488
4.31796	5.169402
3.04056	5.169096
3.72591	5.44584
3.26538	5.008884
3.687216	4.243704
4.014948	4.442562
3.747672	4.797066
2.697036	4.353126
4.932924	5.669262
3.4758	4.741704
4.767984	5.169096
4.89582	5.159664
4.623384	5.564466
4.553844	5.366028
4.623384	6.368082
3.888468	5.357382
3.794352	6.193284
